# Phase II trial of VEGFR2 inhibitor apatinib for metastatic sarcoma: focus on efficacy and safety

**DOI:** 10.1038/s12276-019-0221-7

**Published:** 2019-02-28

**Authors:** Zhichao Liao, Feng Li, Chao Zhang, Lei Zhu, Yehui Shi, Gang Zhao, Xu Bai, Shafat Hassan, Xinyue Liu, Ting Li, Peipei Xing, Jun Zhao, Jin Zhang, Ruwei Xing, Sheng Teng, Yun Yang, Kexin Chen, Jilong Yang

**Affiliations:** 1 Departments of Bone and Soft Tissue Tumor, Tianjin Medical University Cancer Institute & Hospital, Tianjin, 300060 People’s Republic of China; 2 National Clinical Research Center of Cancer, Tianjin Medical University Cancer Institute & Hospital, Tianjin, 300060 People’s Republic of China; 3 Departments of Molecular Imaging, Tianjin Medical University Cancer Institute & Hospital, Tianjin, 300060 People’s Republic of China; 4 Pharmacological Research Center, Tianjin Medical University Cancer Institute & Hospital, Tianjin, 300060 People’s Republic of China; 5 Departments of Pathology, Tianjin Medical University Cancer Institute & Hospital, Tianjin, 300060 People’s Republic of China; 6 Departments of Radiation Oncology, Tianjin Medical University Cancer Institute & Hospital, Tianjin, 300060 People’s Republic of China; 7 Departments of Epidemiology and Biostatistics, Tianjin Medical University Cancer Institute & Hospital, Tianjin, 300060 People’s Republic of China

**Keywords:** Translational research, Sarcoma

## Abstract

Apatinib (YN968D1) is a novel tyrosine kinase inhibitor targeting vascular endothelial growth factor receptor 2 (VEGFR-2). We conducted a single-arm, nonrandomized phase II study (NCT03121846) to assess the efficacy and safety of apatinib in patients with stage IV sarcoma. We recruited 64 patients with stage IV sarcoma who had failed chemotherapy. The primary endpoint was progression-free survival (PFS), and the secondary endpoints were progression-free survival rate (PFR), objective response rate (ORR), and disease control rate (DCR) at week 12. Treatment-related adverse effects (AEs) were evaluated. Fifty-nine patients were assessed for efficacy and 64 patients for AEs. The median PFS was 7.93 months. At 12 weeks, the PFR was 74%, the ORR was 16.95% (10/59), and the DCR was 86.44% (51/59). The final ORR was 15.25% (9/59) and the DCR was 57.63% (34/59). Notably, 22 patients (34.38%) who developed hypertension, hand-foot-skin reaction, or proteinuria had significantly longer OS than those without these AEs (18.20 vs. 10.73 months; *P* = 0.002). We conclude that apatinib is effective and well tolerated in patients with advanced sarcoma. The development of hypertension, hand-foot-skin reaction, or proteinuria may indicate a favorable prognosis, representing a novel finding in sarcoma patients.

## Cancer: Starving sarcomas into submission

A drug that inhibits blood vessel growth offers a potentially promising treatment for a class of tumors with a poor prognosis. Sarcomas form in bone and connective tissue, and patients with advanced disease have a five-year survival rate of less than 10%. Researchers led by Jilong Yang of the Tainjin Medical University Cancer Institute & Hospital in China tested apatanib, a drug that starves tumors by preventing blood vessel development, in late-stage sarcoma patients. Strikingly, 15% of the patients experienced tumor reduction after treatment, and more than half overall achieved at least partial disease control. Adverse events were generally mild, but Yang and colleagues observed that patients who experienced certain side-effects achieved a greater survival benefit from treatment. These results support further investigation of this drug, and offer hints of possible biomarkers to predict response.

## Introduction

Sarcoma is a rare tumor that accounts for ~1% of all adult and 15% of all pediatric malignancies^1,2^. An estimated 16,490 people will be diagnosed with bone and soft tissue sarcomas (STS) in the US in 2018, and ~6740 will die of this disease^2^. The National Central Cancer Registry of China estimated that there were 28,000 new bone sarcoma diagnoses and 20,700 deaths from bone sarcoma in China in 2015^3^. The prognosis of sarcoma patients in stage IV is poor, with a median overall survival (mOS) time for STS of ~12 months and a 5-year survival rate of <10%^4–6^. Patients with advanced bone sarcomas such as osteosarcoma and Ewing’s sarcoma/peripheral neuroectodermal tumor also have a very poor prognosis^7,8^. Although chemotherapy is widely used to treat metastatic sarcomas, conventional chemotherapeutic agents such as ifosfamide, doxorubicin, methotrexate, cisplatin, dacarbazine, gemcitabine, and docetaxel are not curative^9,10^, and combined or dose-dense regimens have largely failed to improve response rates^11,12^. Furthermore, the long-term use of cytotoxic drugs increases the risk of adverse events (AEs). For example, cumulative-dose and dose-intense doxorubicin cause cardiomyopathy, with an associated mortality risk^13,14^. New therapies for metastatic sarcomas are therefore urgently needed.

Angiogenesis is a key process in tumor growth and metastasis, and antiangiogenic agents are an important component of modern tumor therapy^15^. Apatinib (YN968D1) is an orally administered, small-molecule receptor tyrosine kinase inhibitor with potential antiangiogenic and antineoplastic activities^16^. It binds selectively to and inhibits vascular endothelial growth factor receptor 2 (VEGFR-2), and may inhibit VEGF-stimulated endothelial cell migration and proliferation, and decrease tumor microvascular density^17,18^. Phase I–III trials of apatinib have demonstrated encouraging antitumor activity and manageable toxicities in patients with gastric cancer, breast cancer, and non-small-cell lung cancer^16,19–21^.

Clinical investigations of apatinib in metastatic sarcomas have also shown encouraging results. Seven case reports of the use of apatinib to treat sarcoma have been published, and all seven patients were considered to have partial responses (PR)^22–27^, indicating that apatinib can be effective for treating malignant sarcomas, with manageable AEs. Three retrospective studies of apatinib for the treatment of sarcomas have also been conducted to date, including one by our group^28–30^. Yang et al. showed a better objective response rate (ORR) than Li et al. (33.3% vs. 20%), but a poorer median progression-free survival (mPFS; 4.3 months vs. 8.84 months) and disease control rate (DCR; 75.0% vs. 80.0%)^28,29^. Xie and colleagues found that 62.5% of patients with sarcoma achieved PR and 19.6% achieved stable disease (SD), with 36.5% PFS at 6 months and a mOS of 9.9 months^30^. These results suggest that apatinib may represent a promising treatment option for patients with metastatic sarcomas, although its efficacy is currently inconsistent.

These previous retrospective studies used small patient cohorts and only supported a preliminary role for apatinib in the treatment of sarcoma. Interestingly, one study found that the presence of hypertension (HTN), proteinuria, or hand-foot syndrome (HFS) during the first cycle of apatinib treatment correlated with better outcomes in patients with gastric cancer and was a viable biomarker of antitumor efficacy in patients with metastatic gastric cancer^31^. However, no such phenomenon has been observed in sarcoma patients, possibly because of the small patient cohorts. We therefore conducted a single-arm, phase II study (NCT03121846) to assess the efficacy and toxicity of apatinib in patients with stage IV sarcoma who had failed conventional therapies.

## Materials and Methods

### Patients, ethical clearance, study drug dosing and treatment

This single-arm phase II trial (NCT03121846) was designed to assess the biological activity of apatinib in relation to its efficacy and safety. The study was conducted in accordance with the Declaration of Helsinki and was approved by the Institutional Review Board or Ethics Committee at each participating center. All patients gave written informed consent.

PFS and ORR were the main therapeutic indices in this clinical study. Assuming a two-sided distribution, the test efficiency was 0.80. Previous studies reported an ORR of 6% for pazopanib in patients with stage IV STS^5,6,32–34^. Our preliminary results and reported data showed that the ORR of apatinib was approximately 20%^28–30,35^. A required sample size of 54 was calculated according to the sample formula (calculated by PASS software). Taking account of possible patient dropouts (estimated as 10%), the required number of cases was 59. We therefore set the sample size as 60–80.

We recruited 64 patients with stage IV sarcomas who failed prior chemotherapy and treated them with apatinib monotherapy from September 2015 to February 2018. All patients received apatinib at a starting dose of 500 mg/day on days 1–28 of each 4-week cycle. Doses could be reduced twice, to 375 mg and then 250 mg if necessary. Patients who could not tolerate the 250 mg dose were excluded from the trial.

### Efficacy

Pretreatment evaluation included physical examination, clinical blood counts and blood chemistry, and computed tomography scans of measurable lesions at baseline. Toxicity was assessed monthly. Measurable lesions were assessed by computed tomography after every two cycles (8 weeks), or more often in patients who showed evidence of substantial progression, or in patients who quit the trial. Patients were observed until death, loss to follow-up, quitting the trial, or the end of the study.

Clinical benefit responses were evaluated according to the Response Evaluation Criteria in Solid Tumors 1.1 (RECIST 1.1)^36^. Evidence of efficacy was agreed upon by two independent radiologists who were blinded to the treatment. Each patient had at least one measurable extracranial lesion, and responses were evaluated according to RECIST 1.1^37,38^. Based on benefits in previous retrospective studies of apatinib in sarcoma patients^25,27–30,39^, some sarcoma patients with nonmeasurable lesions were also enrolled and evaluated according to RECIST 1.1^37,38^. Nonmeasurable lesions included small lesions (longest diameter < 10 mm, or pathological lymph nodes with 10–15 mm short diameter) as well as truly nonmeasurable lesions. Lesions considered truly nonmeasurable included leptomeningeal disease, ascites, pleural or pericardial effusion, lymphangitic involvement of skin or lung, or abdominal mass/abdominal organomegaly identified by physical exam but not measurable by reproducible imaging techniques^36,38^. Patients with these nonmeasurable lesions were evaluated as CR, progressive disease (PD), or non-CR/non-PD according to RECIST 1.1^36,38^. Non-CR/non-PD was preferred over SD for nontarget diseases^37,38^. To simplify and unify the evaluation, we replaced non-CR/non-PD with SD in some patients with nonmeasurable lesions.

Because this trial (NCT03120846) was designed to assess the biological activity and side effects of apatinib, we considered PFS as the primary endpoint, with progression-free rate (PFR), ORR, and DCR at 12 weeks as secondary endpoints. PFS was defined as time from initiating apatinib treatment until disease progression according to RECIST 1.1. Disease control was defined as complete response (CR), PR, or SD. The ORR was (CR + PR) / total number of cases × 100%. The DCR was (CR + PR + SD) / total number of cases × 100%.

### Safety and toxicity assessments

All patients who received at least one dose of apatinib were included in the safety and toxicity analyses. Treatment-related AEs were assessed and graded based on the National Cancer Institute Common Terminology Criteria for Adverse Events (CTCAE, version 3.0)^40^.

### Statistical analysis

All statistical analyses were conducted using the Statistical Package for the Social Sciences software (SPSS) version 20.0. Quantitative variables were compared among groups using analysis of variance or Kruskal–Wallis tests. Pearson’s *χ*^2^ test or Fisher’s exact test was used to analyze categorical variables, and the Kruskal–Wallis test was used to analyze ordered variables. We compared PFS and OS between the different groups using a log-rank (Mantel–Cox) test with a Cox proportional hazards model to estimate hazard ratios and to test for significance. We also evaluated differences in PFS and OS between groups using the multiple Cox model. ORR and DCR analyses were based on frequencies. All statistical analyses were two-sided, and significance was set at *P* < .05 or at the 95% confidence interval for the results of other statistical tests.

## Results

### Patient demographics

We recruited 64 patients with stage IV sarcomas in this trial (33 males, 31 females, average age 42.16 years, range 11–83 years) from September 2015 to February 2018 (Table 1). Their average Eastern Cooperative Oncology Group (ECOG) performance status was 1.55 (0–2; two patients were evaluated as 3). The pathological types of the 22 bone sarcomas included osteosarcoma (*n* = 11), Ewing’s sarcoma/peripheral neuroectodermal tumor (*n* = 7), chordoma (*n* = 2), and chondrosarcoma (*n* = 2), and the pathological types of the 42 STSs included undifferentiated pleomorphic sarcoma (*n* = 6), malignant peripheral nerve sheath tumor (*n* = 7), synovial sarcoma (*n* = 6), leiomyosarcoma (*n* = 5), fibrosarcoma (*n* = 5), rhabdomyosarcoma (n = 6), and other STS (*n* = 7). All patients had stage IV disease according to American Joint Committee on Cancer staging. Metastatic sites included lung, liver, bone, lymph node, and soft tissues, with lung being the most common metastatic site (Table 1). The clinicopathological characteristics had no significant effect on survival, except for ECOG performance status, which significantly affected PFS (log rank = 4.791, *P* = 0.029; Table 1).

**Table 1:** Clinicopathological characteristics of patients with sarcoma treated with apatinib

Characteristic	Value
Age	
Mean	42.16 yr
Range	11–83 yr
Distribution	
= <42	31(48.4%)
>42	33(51.6%)
Sex	
Male	33(51.6%)
Female	31(48.4%)
ECOG performance-status score	
0	2(3.1%)
1	27(42.2%
2	33(51.6%)
3	2(3.1%)
Tumor type-no (%)	
Bone sarcomas	22(34.4%)
Osteosarcoma	11(17.3%)
Chondrosarcoma	2(3.1%)
PNET/EWS	7(10.9%)
Chordoma	2(3.1%)
Soft tissue sarcomas	42(65.6%)
UPS	6(9.4%)
Synovial sarcoma	6(9.4%)
MPNST	7(10.9%)
LMS	5(7.8%)
RMS	6(9.4%)
Fibrosarcoma	5(7.8%)
Other sarcoma	7(10.9%)
Metastasis site	
Lung	42(65.7%)
Lung and other sites	15(23.4%)
Nonlung metastasis	7(10.9%)

*ECOG* Eastern Cooperative Oncology Group

The follow-up time at the data analysis date (February 28, 2018) was 0–28 months. The PFS time was 0.93-–2.63 months (mean 5.19 months), and the OS was 0.93–28.00 months (mean 7.42 months).

### Maximum change in target lesion size

**Fig. 1:**
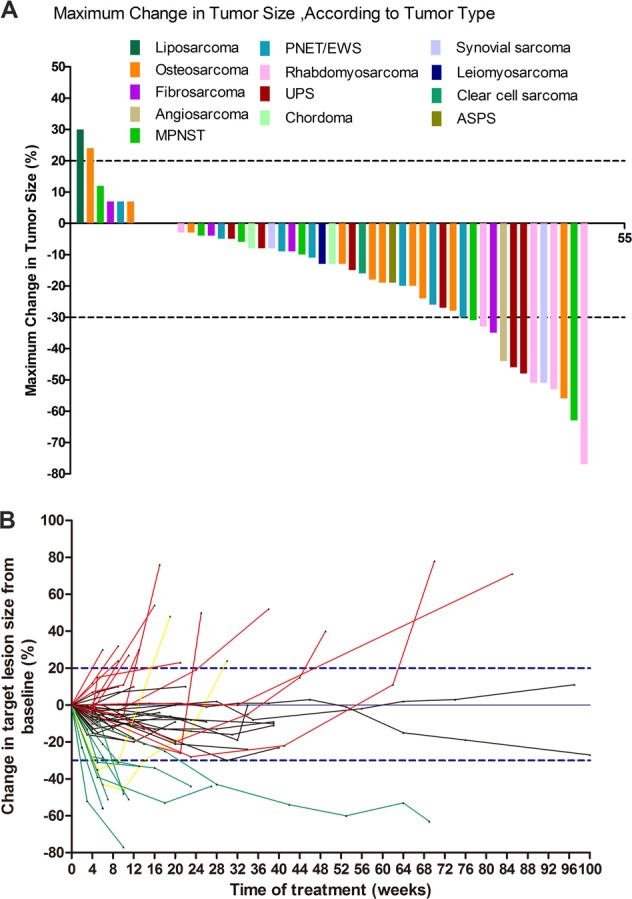
Maximum changes and all changes from baseline in target lesions in patients with stage IV sarcomas treated with apatinib. **a** Maximum changes in target lesions in patients with stage IV sarcoma treated with apatinib. Among 64 patients, 51 were evaluated for response to apatinib (RECIST 1.1). No patients achieved CR, 13 (25.49%) achieved PR once, and 36 (70.59%) achieved SD once. Only two (3.9%) patients suffered from PD, and 49 (96.1%) responded to apatinib monotherapy. **b** Changes from baseline in target lesions after apatinib treatment in 51 patients with measurable sarcoma lesions. Green lines: target lesions shrank ≥ 30% from baseline; red lines: target lesions increased ≥ 20% from baseline; yellow lines: target lesions initially decreased ≥ 30% and then increased ≥ 20% from baseline; black lines: target lesions changed from 20%–30%

Maximum change in target lesion size was evaluated according to RECIST 1.1, including data for 51 patients (Fig. 1A). No patients achieved CR. However, 13 patients (25.49%) achieved PR, 36 (70.59%) achieved SD, and two (3.9%) suffered from PD at the first evaluation. Thus, up to 96.1% (49/51) of patients had some response to apatinib monotherapy (Fig. 1A).

There was no significant difference in maximum change in tumor size between bone sarcomas and STS (Supplemental Fig. 1A, B). All rhabdomyosarcoma and undifferentiated pleomorphic sarcoma tumors decreased significantly at some point during apatinib treatment, while the sizes of osteosarcomas, malignant peripheral nerve sheath tumors, and Ewing’s sarcoma/peripheral neuroectodermal tumors showed no significant increase or decrease during treatment (Supplemental Fig. 1C, D).

### Clinical responses at 12 weeks

At 12 weeks, 59 patients had received at least one full treatment cycle and were included in our efficacy evaluation. Five patients had received less than one cycle and were only included in the safety evaluation (Table 2).

**Table 2:** Clinical response to apatinib in patients with metastatic sarcoma

Response	12 *W*	Overall response
CR	0	0
PR	10	9
SD	41	25
PD	8	25
Excluded	5	5
ORR	16.95% (10/59)	15.25% (9/59)
DCR	86.44% (41/59)	57.63% (34/59)
	PFR-_12W_ = 74%, OSR_-12W_ = 92%	mPFS = 7.93 m, mOS = 17.27 m

*CR* complete response, *PR* partial response, SD stable disease, *PD* progressive disease, *DCR* disease control rate, *ORR* objective response rate, *PFR* progression-free survival rate, *mPFS* median progression-free survival

Of the 59 evaluated patients, none achieved CR, 10 achieved PR, 41 achieved SD, and eight patients suffered from PD (Table 2). The ORR at 12 weeks was 16.95% (10/59), the DCR was 85.44% (51/59), the PFR was 74%, and the OS rate was 92% (Table 2).

Regarding the different sarcoma types, there was a significant difference in ORR at 12 weeks between bone sarcomas and STS, because all patients with PR had STS (0 [0/21] vs. 26.32% [10/38], Fisher’s exact test, *P* = 0.022) (Supplemental Table 1). However, the DCR, PFR, and OS rate at 12 weeks did not differ significantly between bone sarcomas and STS (Supplemental Table 1).

### Overall response

Sixty-four patients were enrolled in this trial by February 28, 2018 (Table 1), of whom five had received less than one cycle and were only included in the safety evaluation. Fifty-nine patients were included in the final efficacy evaluation (Table 2), including 51 patients with at least one measurable extracranial lesion and eight patients with nonmeasurable lesions.

**Fig. 2:**
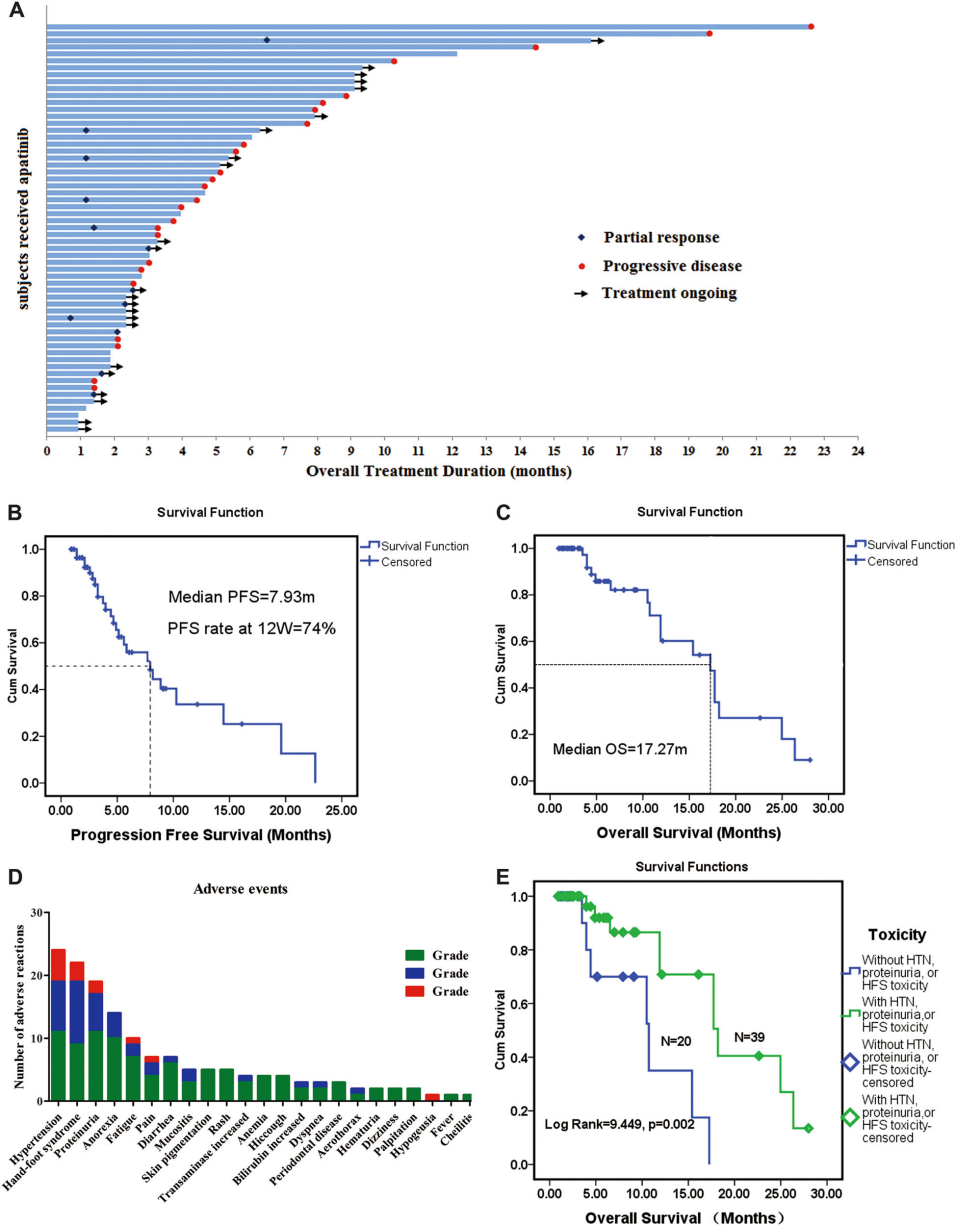
Efficacy and toxicity of apatinib in sarcoma patients. **a** Overall responses of 59 patients with stage IV sarcoma treated with apatinib. Among 59 patients, 51 had measurable lesions and eight patients had unmeasurable lesions. Responses were PR in nine (15.25%), SD in 25 (42.37%), and PD in 25 (42.37%). **b**, **c** PFS and OS in 59 patients treated with apatinib. **b** Median PFS was 7.93 months; PFR at 12 weeks was 74%. **c** Median OS was 17.27 months. **d**, **e** Frequency and prognostic role of apatinib toxicity in sarcoma patients. **d** Severe adverse events (AEs) included no grade 4 AEs and grade 3 AEs in nine patients (14.06%), mainly hypertension (HTN), hand-foot syndrome (HFS), proteinuria, fatigue, and dysgeusia. **e**: HTN, proteinuria, and HFS were significantly correlated with longer OS in this cohort, and patients who suffered from any of these three AEs during treatment had significantly longer OS than those without these AEs

We calculated changes in target lesion size from baseline in the 51 patients with measurable lesions (Fig. 1B). The responses according to RECIST 1.1 at the final evaluation were PR in nine (15.25%, 9/59), SD in 25 (42.37%, 25/59), and PD in 25 (42.37%, 22/52) (Table 2, Fig. 2A), giving a final overall ORR of 15.25% (9/59) and final DCR of 57.69% (34/59) (Table 2). The median PFS as the primary endpoint was 7.93 months (Fig. 2B), and the median OS was 17.27 months (Fig. 2C).

We also evaluated the relationships between apatinib response and sarcoma type. The ORR differed significantly between bone sarcomas and STS, because all PR patients had STS (0 [0/21] compared with only 23.68% [9/38] with bone sarcoma; Fisher’s exact test, *P* = 0.034; Supplemental Table 1). However, DCR, mPFS, and mOS did not differ significantly between bone sarcomas and STS (Supplemental Table 1 and Supplemental Fig. 2A, B).

### Long-term responses

The PFR at 12 weeks was 74% for sarcoma patients treated with apatinib, and the PFRs at 6 months, 9 months, and 1 year were 56%, 40%, and 34%, respectively, with no significant drop-off from 12 weeks (Fig. 2B).

**Fig. 3:**
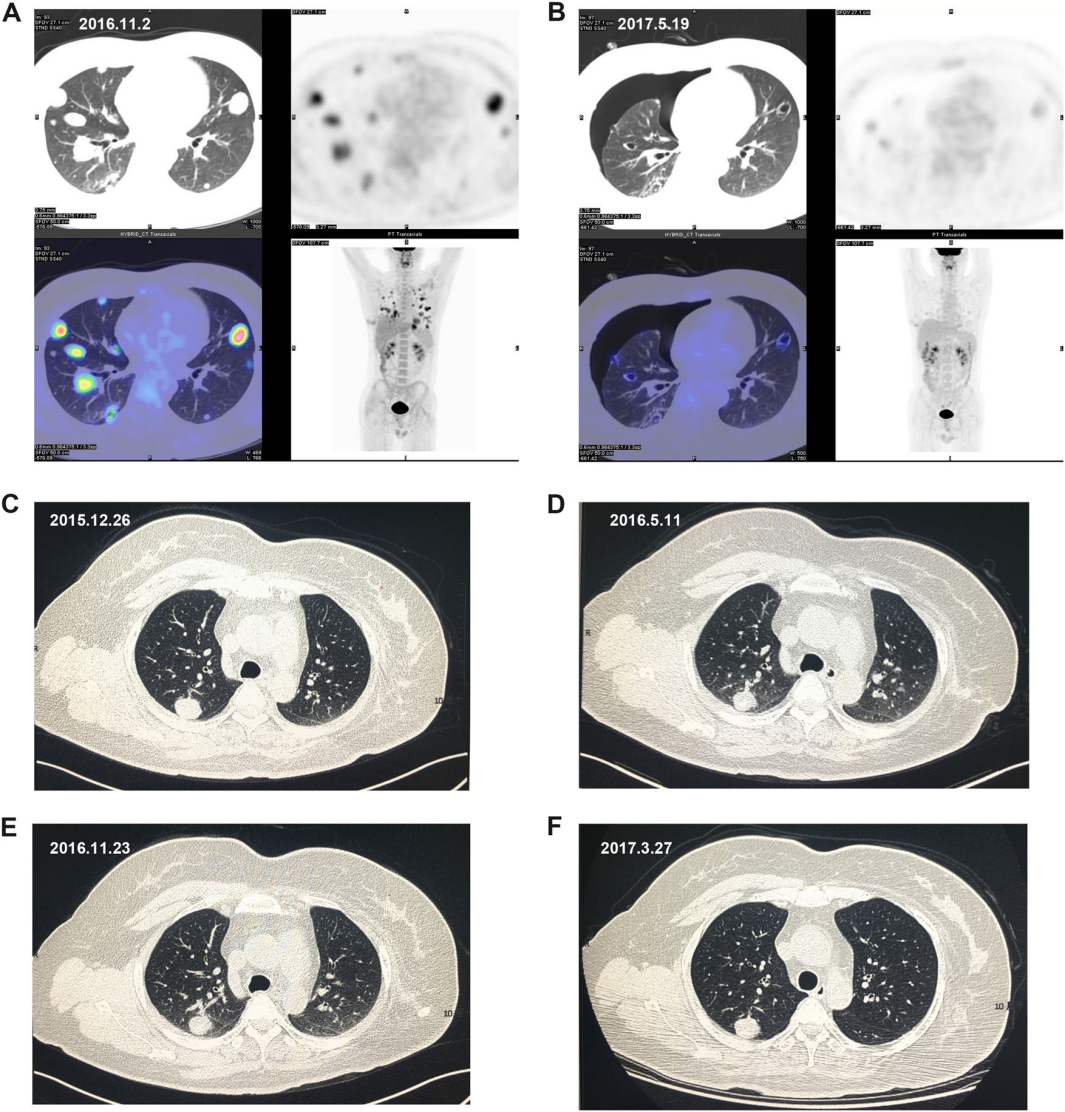
Typical responses to apatinib treatment in two sarcoma patients. **a**, **b** Metastatic undifferentiated pleomorphic sarcoma lesions showed significant decrease in size and pulmonary cavities after apatinib treatment. Positron emission tomography-computed tomography (PET-CT) showed decreased tumor size, metabolic activity, pulmonary cavities, and severe pneumothorax after apatinib treatment. **a** Before apatinib treatment (2016.11.2). **b** After apatinib treatment (2017.5.19). **c**–**f** Stable lung metastatic lesion in patient with synovial sarcoma treated with apatinib. Chest CT scan showed long-term stable disease. **c**: CT scan before treatment (2015.12.26). **d** CT scan after treatment (2016.5.11). **e** CT scan after treatment (2016.11.23). **f** CT scan after treatment (2017.3.27)

Twelve patients achieved long-term responses by the median PFS (7.93 months, 32.7 weeks), and four of these were still responsive to the drug after 12 months (Fig. 2a). One of these four patients suffered from PD at 14.47 months (62 weeks); another female patient with leiomyosarcoma achieved long-term SD for 24 months, but then switched to anti-PD-L1 therapy; one patient with a PR of metastatic undifferentiated pleomorphic sarcoma quit the trial at 16.1 months (69 weeks; Fig. 3A, B); and one patient with metastatic synovial sarcoma in her lung achieved SD for 22.3 months, but developed grade II HFS, grade II proteinuria, and grade I HTN (Fig. 3c–f).

Twenty-three patients were still receiving apatinib at the data analysis date (February 28, 2018). Forty-one patients (64.06%) were off protocol, of whom 13 (31.70%) received postprotocol apatinib combined with chemotherapy, four (9.76%) received apatinib combined with the anti-PD-L1 antibody pembrolizumab, and the other 24 patients (58.54%) received best supportive care.

### Safety, toxicity, and their clinical significance

The 64 patients who received at least one dose of apatinib were included in the safety evaluation. Common AEs included HTN (*n* = 24, 37.50%), HFS (*n* = 22, 34.38%), proteinuria (*n* = 19, 29.69%), anorexia (*n* = 14, 21.88%), fatigue (*n* = 10, 15.63%), pain (*n* = 7, 10.94%), diarrhea (*n* = 7, 10.94%), and others (Table 3, Fig. 2D). No grade 4 AEs occurred, but nine patients (14.06%) suffered from grade 3 AEs, which were mainly HTN, HFS, proteinuria, fatigue, pain, and dysgeusia (Table 3, Fig. 2D).

**Table 3:** Adverse events in 64 patients with sarcoma treated with apatinib

Adverse events^a^	Grade 1	Grade 2	Grade 3	Total
*Hypertension*	11	8	5	24(37.50%)
*Hand-foot syndrome*	9	10	3	22(34.38%)
*Proteinuria*	11	6	2	19(29.69%)
Anorexia	10	4		14(21.88%)
Fatigue	7	2	1	8(12.50%)
Pain	4	2	1	7(10.94%)
Diarrhea	6	1		7(10.94%)
Mucositis	3	2		5(7.81%)
Skin pigmentation	5			5(7.81%)
Rash	5			5(7.81%)
Transaminase increased	3	1		4(6.25%)
Anemia	4			4(6.25%)
Hiccough	4			4(6.25%)
Bilirubin increased	2	1		3(4.69%)
Dyspnea	2	1		3(4.69%)
Periodontal disease	3			3(4.69%)
Aerothorax	1	1		2(3.13%)
Hematuria	2			2(3.13%)
Dizziness	2			2(3.13%)
Palpitation	2			2(3.13%)
Hypogeusia			1	1(1.56%)
Fever	1			1(1.56%)

^a^According to CTCAE 4.0

Fifteen (23.44%) patients quit the trial, including eight (12.50%) who quit for personal reasons. One patient (1.56%) quit because of an uncontrolled urinary tract infection, and one (1.56%) because of wound nonunion. Five (7.81%) patients had dose adjustments or quit the trial during treatment because of grade 3 HTN, proteinuria, fatigue, or dysgeusia, including two (3.13%) patients who quit because of grade 3 proteinuria and dysgeusia, respectively. One patient (1.56%) suffered from grade 3 HTN, fatigue, and pain, and his apatinib dose was reduced to 250 mg/day after one cycle, but he quit after two cycles at 250 mg due to uncontrolled HTN. The other two (3.13%) patients stopped treatment for a week and then continued treatment with controlled HTN and proteinuria.

We also evaluated the clinical significance of the AEs. Interestingly, although the presence of these AEs showed no correlation with the clinical response signatures to apatinib treatment, such as ORR and DCR, the 22 patients (34.38%) who suffered from HTN, HFS, or proteinuria during treatment showed significantly better OS than those without these AEs (18.20 vs. 10.73 months; log rank = 9.449, *P* = 0.002; Table 3, Fig. 2E). However, these AEs showed no prognostic value for PFS (Supplemental Fig. 2C). When we excluded two patients who suffered from HTN at day 30 and HFS at day 32, respectively, the results for the remaining 20 patients were similar to those for the other patients (OS: 18.20 vs. 10.73 months; log rank = 9.676, *P* = 0.002; Supplemental Fig. 2D).

## Discussion

Sarcomas are malignant mesenchymal tumors with unique clinical and histologic features, comprising more than 50 subtypes^41^. Although sarcomas are less common than other epithelial tumors, they account for almost 21% of all solid tumors in children and are the third leading cause of cancer-related death among people under 20 years old^2,41^. Despite recent significant developments in multimodal therapies, the 5-year survival rate has remained relatively unchanged^42^. This is particularly evident in patients with metastatic or recurrent advanced disease, who have a median OS of ~12 months, with only 10% of patients remaining alive at 5 years^43^. Novel strategies and innovative therapies for patients with sarcomas are therefore urgently needed.

Overexpression of VEGFRs, particularly VEGFR-2, has been significantly associated with low survival rates in patients with sarcomas^44–48^, and VEGF/VEGFR-targeted therapy is thus indicated for sarcoma based on its effects on angiogenesis. Apatinib is an orally administered, small-molecule receptor tyrosine kinase inhibitor with potential antiangiogenic and antineoplastic activities^16^. Liu et al. showed that apatinib inhibited osteosarcoma growth both in vivo and in vitro^48^. Apatinib inhibited osteosarcoma growth in vitro by inducing autophagy and apoptosis of osteosarcoma cells via directly inhibiting expression of the antiapoptotic protein Bcl-2 and inactivating signal transducer and activator of transcription 3 (STAT3), which is mediated by VEGFR2^48^. Interestingly, the effect of apatinib on apoptosis in osteosarcoma cells was enhanced by inhibiting autophagy^48^. Zheng et al. showed that apatinib attenuated migration and invasion by suppressing epithelial–mesenchymal transition and inactivating STAT3. Furthermore, apatinib reduced PD-L1 expression in osteosarcoma cells^49^. These data suggest that apatinib could work as both a targeted therapeutic drug and an immunotherapy modulator in sarcoma patients. Apatinib has also demonstrated efficacy in several case reports and retrospective studies of patients with malignant sarcomas, with manageable AEs^22–26,28,29^.

We conducted the first phase II clinical trial to evaluate the efficacy and safety of apatinib in the largest cohort of patients with metastatic sarcoma to date. The results indicated that apatinib was effective for treating sarcoma, based on the mPFS (7.93 months), PFR (74%), ORR (13.95%), and DCR (81.39%) at 12 weeks. The final ORR rate was 15.25% (9/59), and the final DCR was 57.69% (34/59). These results were in accord with the previously reported efficacy of apatinib for sarcomas, and with case reports and small-cohort retrospective studies^23–26,28,29,39,48–50^. The efficacy demonstrated in these studies was also comparable to that of other single-agent angiogenesis inhibitors, such as pazopanib, sunitinib, sorafenib, and anlotinib^34,51–55^, although no direct comparative studies have yet been conducted. Heudel et al. enrolled 246 patients with metastatic STS in a phase III trial of pazopanib, of whom 14 achieved PR, 164 SD, and 57 PD; the ORR was 6.0% (14/246) and the DCR was 72.4% (178/246)^51^. A phase II, multicenter clinical trial of sunitinib in 53 patients with advanced nongastrointestinal stromal STS was carried out by George et al., with one patient achieving confirmed PR and 10 patients (20%) achieving SD for at least 16 weeks^54^. A phase II clinical study of sorafenib for metastatic or recurrent sarcoma with a follow-up time of 6 months demonstrated PFS of 3.2 months^53^. Recent reports of another targeted therapeutic drug, anlotinib, showed that the PFR at 12 weeks was 68% and the ORR was 13% (95% confidence interval 7.6%–18%). The median PFS and OS were 5.6 and 12 months, respectively^52,55^. Apatinib thus shows encouraging efficacy for the treatment of metastatic sarcomas compared with these other drugs, with manageable toxicity.

The present study also suggested several other intriguing points. First, our data indicated that the short-term effect of apatinib was good, with 96.1% of sarcoma patients initially responding to apatinib monotherapy. Although no patient had a CR, 25.49% of all our sarcoma patients had PR, and more than 70.59% patients showed SD at some point, while only 3.9% patients suffered from PD at their first evaluation. Second, some patients had long-term clinical responses, with nine patients evaluated as PR (15.25%, 9/59), 25 as SD (42.37%, 25/59), and 25 as PD (42.30%, 22/52) at the end of the follow-up period. The final overall ORR of 15.25% and DCR of 57.63% were similar to the clinical responses at 12 weeks. Furthermore, the PFR at 6 months, 9 months, and 1 year were 56%, 40%, and 34%, respectively, with no significant drop-off from the PFR of 74% at 12 weeks. Twelve patients had long-term responses in terms of mPFS (7.93 months, 32.7 weeks), four of whom were still responding to the drug after 12 months. One female patient with leiomyosarcoma achieved long-term SD for 24 months but then switched to anti-PD-1 therapy. Third, patients with metastatic STS showed significantly higher ORR than those with bone sarcomas, although there was no significant difference in DCR, mPFS, or mOS between these two sarcoma types.

The most commonly reported AEs of targeted antiangiogenesis agents include HFS, HTN, proteinuria, rash, diarrhea, hyperbilirubinemia, rash/desquamation, fatigue, thrombocytopenia, leukopenia, diarrhea, nausea, and vomiting^34,53,54^. The most frequently observed AEs associated with apatinib were HFS, proteinuria, and HTN, which are similar to those reported in a phase I study of apatinib in patients with metastatic gastric cancer and in retrospective studies in sarcoma patients^29,39,56,57^. There were no grade 4 AEs in the present study, although 14.06% of patients suffered from grade 3 AEs, mainly HTN, HFS, proteinuria, fatigue, pain, and dysgeusia. The frequencies of these AEs in the present study were similar to other data for sarcomas^25,28–30^. HTN could be well controlled by angiotensin receptor blockers with or without calcium antagonists (such as amlodipine), in addition to dose interruption or reduction. Hematologic toxicities, including neutropenia and thrombocytopenia, were mild to moderate, and no dose interruption or reduction was needed during the current treatment. These results indicated that apatinib is well tolerated, although HTN, HFS, and proteinuria should be monitored.

The side effects of apatinib are manageable, in addition to which patients with HTN, proteinuria, or HFS had significantly longer OS than patients without any of these AEs, similar to the results for gastric cancers^31,58,59^. Although the presence of these AEs did not correlate with clinical response signatures such as ORR and DCR, these results provide the first evidence for a prognostic value of these manageable toxicities in patients with sarcomas. However, the reasons for this association remain unknown. As an orally administered, small-molecule receptor tyrosine kinase inhibitor, apatinib has potential antiangiogenic and antineoplastic activities, and has been shown to inhibit VEGFR2 and regulate PD-L1 expression, apoptosis, autophagy, and epithelial–mesenchymal transition. However, the true mechanisms of apatinib in sarcoma treatment and the long-term effect of this treatment remain unclear. We therefore suggest that apatinib might alter the tumor microenvironment, thus affecting AEs and OS. However, further studies are needed to clarify the relationship between OS and the severity of side effects.

Other issues also need to be clarified, including the sensitivities of different types of sarcomas to apatinib treatment. Even patients with metastatic STS showed significantly higher ORRs than did patients with bone sarcomas, although DCR, mPFS, and mOS did not differ significantly between these two sarcoma types. Similarly, several research groups failed to identify any significant differences in sensitivity to apatinib among different sarcoma types, although leiomyosarcomas, rhabdomyosarcomas, and osteosarcomas tended to be more sensitive^22–30,49^. These results might reflect the diverse sarcoma types and the small cohorts in some studies. Furthermore, there is currently no feasible biomarker for predicting response to apatinib treatment, even though apatinib can reportedly inhibit VEGFR2 and regulate PD-L1 expression, apoptosis, autophagy, and epithelial–mesenchymal transition^31,48,49,56,60^. The mechanism of apatinib treatment in sarcomas is thus unclear, and its elucidation might help in the selection of more sensitive predictors for sarcoma patients.

Overall, the results of this phase II clinical trial in a large cohort confirmed that apatinib represents an effective and well-tolerated antiangiogenesis-targeted drug for the treatment of sarcomas. Notably, the occurrence of HTN, HFS, and/or proteinuria may predict a favorable prognosis.

In summary, apatinib exhibited encouraging objective efficacy with manageable toxicity in patients with stage IV sarcomas, with favorable PFS, ORR, DCR, and PFR. In addition, the occurrence of HTN, HFS, and proteinuria may indicate a favorable overall prognosis in these patients. Apatinib may thus benefit sarcoma patients, although further randomized controlled trials are needed to further define its activity and safety.

## Supplementary information

Supplemental data
