# Supplementary material for: Phase II trial of VEGFR2 inhibitor apatinib for metastatic sarcoma: focus on efficacy and safety
Source: Exp Mol Med. 2019 Feb 28;51(3):24. doi: 10.1038/s12276-019-0221-7 (PMC6395676; doi:10.1038/s12276-019-0221-7)
Supplement: Supplementary file 1 — Supplemental data [file 12276_2019_221_MOESM1_ESM.doc]

**Supplemental Figure 1. Maximum change in target lesion size in patients with stage IV sarcoma treated with apatinib.**

Maximum change in target lesion size in patients with (**A**) stage IV soft tissue sarcoma (**B)** stage IV bone sarcoma, (**C**) stage IV rhabdomyosarcoma, or (**D)** stage IV undifferentiated pleomorphic sarcoma.

**Supplemental Figure 2. Survival of patients with different sarcomas treated with apatinib.**

There was no significant difference between patients with bone sarcoma and those with soft tissue sarcoma in terms of (**A**) median progression-free survival or (**B**) median overall survival. **C.** PFS was similar between patients who suffered from hypertension (HTN), proteinuria, and/or hand-foot syndrome (HFS) during treatment and those without these adverse events. **D.** Patients who suffered from HTN, proteinuria, and/or HFS in the first 4 weeks of treatment showed significantly longer overall survival than those without these adverse events.

**Supplemental Figure 1**

**A B**

**
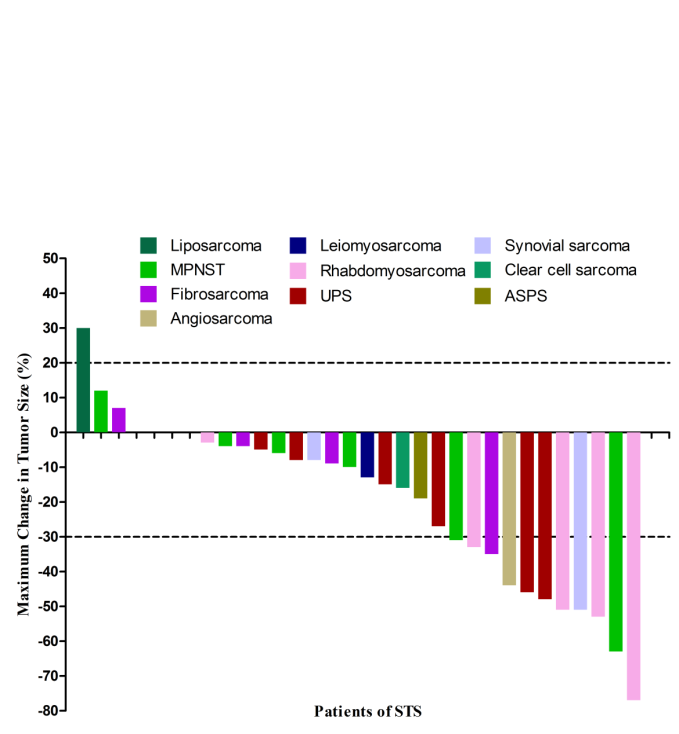

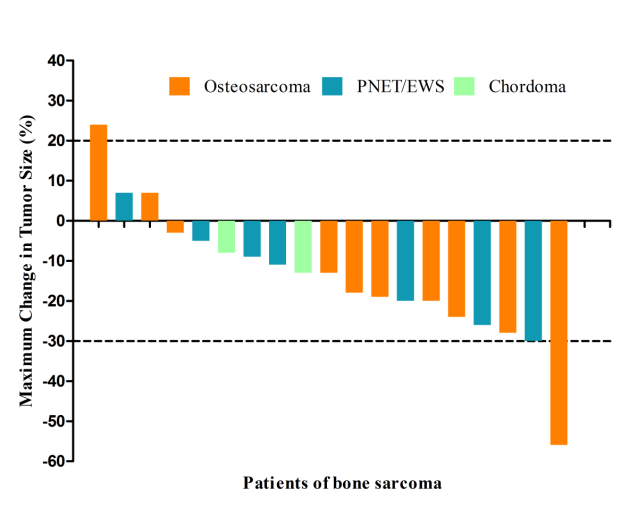
**

**C D**


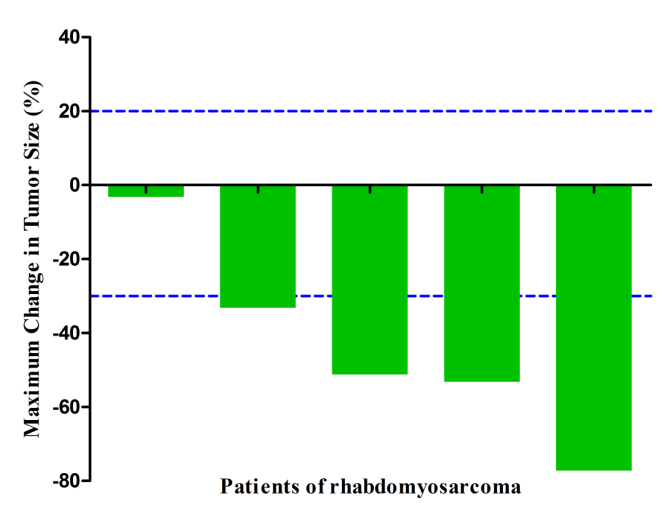
**
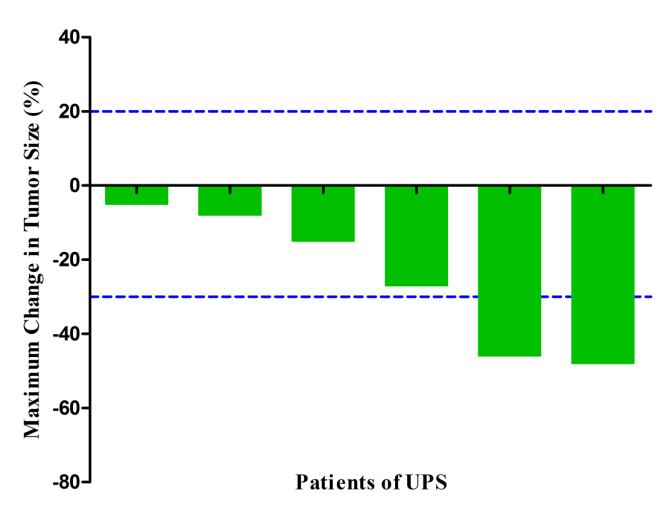
**

**Supplemental Figure 2**

**A B**


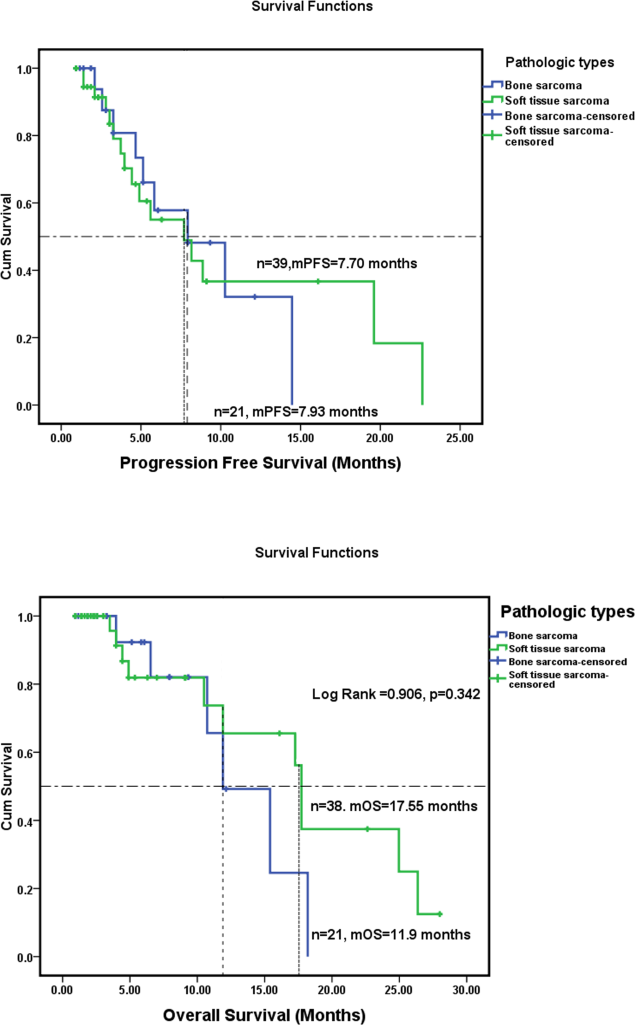

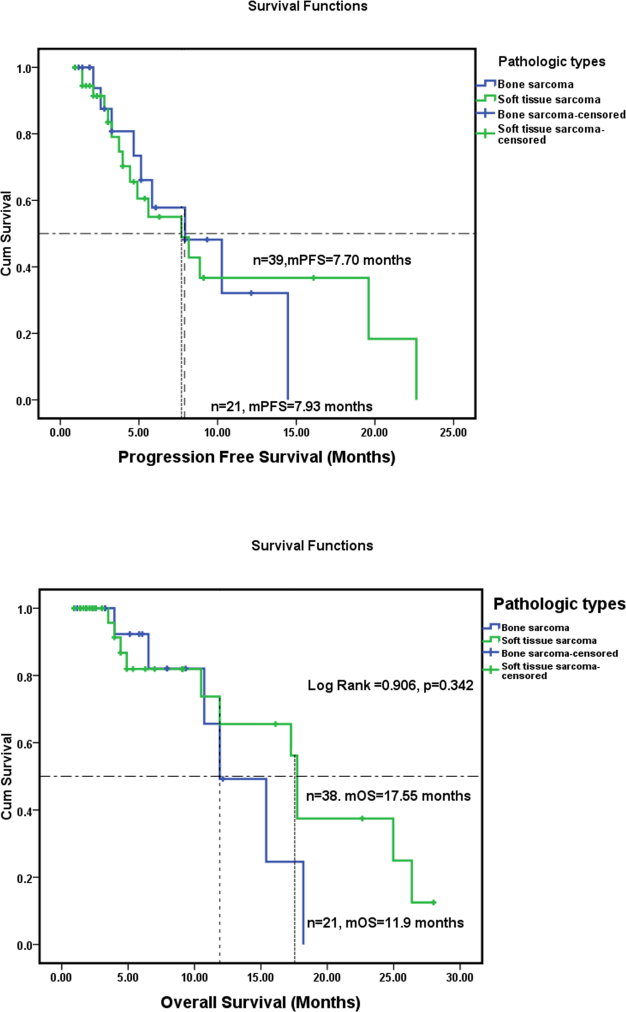


**C D**


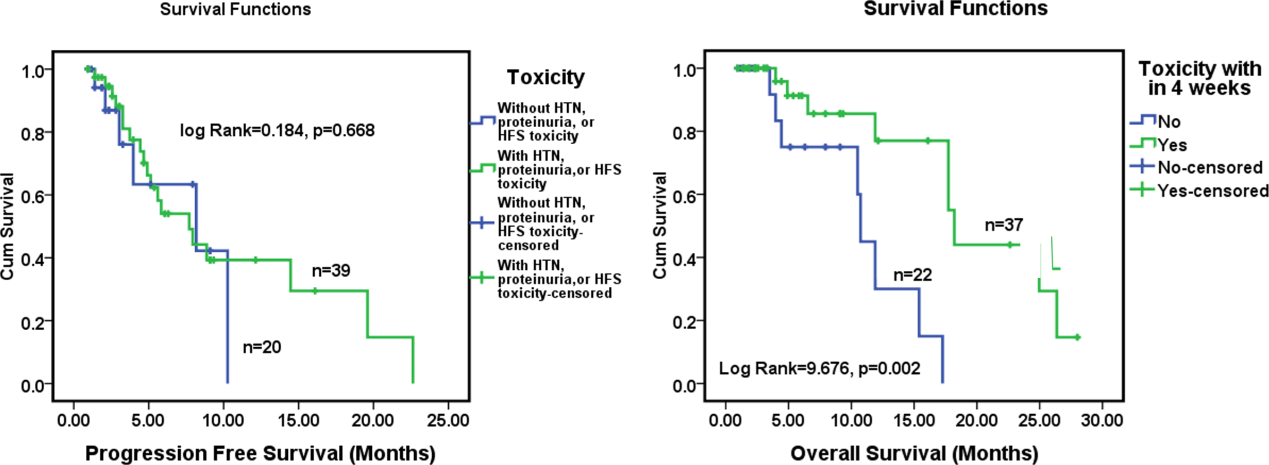

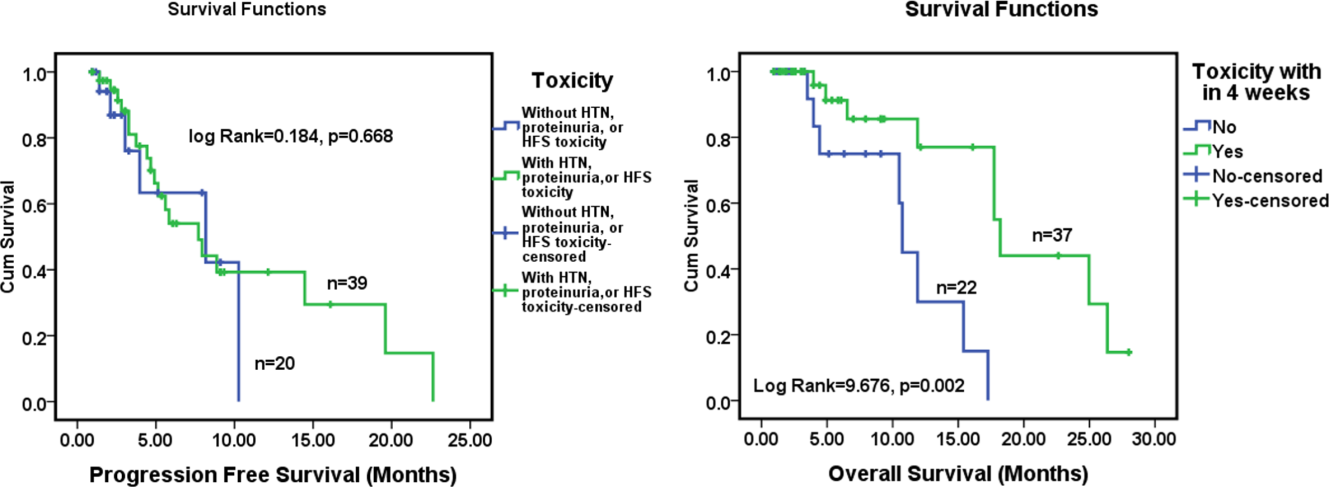


**Supplemental Table 1. Clinical responses to apatinib treatment in patients with bone sarcoma and soft tissue sarcoma.**

| **Response** | **12W** | | **Overall Response** | |
| --- | --- | --- | --- | --- |
|  | Bone sarcomas | Soft tissue sarcomas | Bone sarcomas | Soft tissue sarcomas |
| **CR** | 0 | 0 | 0 | 0 |
| **PR** | 0 | 10 | 0 | 9 |
| **SD** | 18 | 23 | 12 | 13 |
| **PD** | 3 | 5 | 9 | 16 |
| **Excluded** | 1 | 4 | 1 | 4 |
| **ORR** | 0(0/21) | 26.32% (10/38)* | 0(0/21) | 23.68% (9/38)** |
| **DCR** | 85.71%(18/21) | 86.84% (33/38) | 57.14%(12/21) | 57.89% (22/38) |
|  | PFR-_12W_=80%  OSR_-12W_=93% | PFR-_12W_=70%  OSR_-12W_=91% | mPFS=7.71m,  mOS=11.99m | mPFS=7.87m,  mOS=17.55m |

* Fisher’s exact test, *P*=0.022

** Fisher’s exact test, *P*=0.034
